# Deaf and Mute Patient Developing Recurrent Idiopathic Bilateral Optic Neuritis: A Case for Early Treatment With Plasmapheresis?

**DOI:** 10.7759/cureus.10663

**Published:** 2020-09-26

**Authors:** Nicholas T Manasewitsch, Lydia Morrison, Daniel Antwi-Amoabeng, Mihir G Dave, Gurpreet S Chahal

**Affiliations:** 1 Internal Medicine, University of Nevada, Reno School of Medicine, Reno, USA; 2 Internal Medicine, University of Nevada Reno School of Medicine, Reno, USA

**Keywords:** optic neuritis, corticosteroids, mute, deaf, american sign language, multiple sclerosis, plasma exchange, demyelinating, autoimmune, plasmapheresis

## Abstract

Bilateral idiopathic optic neuritis is an uncommon presentation of optic neuritis, and the initial treatment options are limited to corticosteroids with consideration for plasma exchange therapy as second-line therapy. We present the case of a 43-year-old deaf and mute patient whose ability to communicate via American Sign Language was severely impaired by her recurrent idiopathic bilateral optic neuritis. She was treated early and aggressively with both plasma exchange and corticosteroids within days of presentation and experienced rapid improvement in her vision. Early treatment with plasma exchange should be considered in patients whose impairment presents a significant communication barrier.

## Introduction

Optic neuritis is an inflammatory disease characterized by the demyelination of the optic nerve. It most commonly presents unilaterally and is associated with several systemic autoimmune diseases, especially multiple sclerosis (MS) [1]. The recommended therapy for these patients is intravenous (IV) methylprednisolone as per the landmark Optic Neuritis Treatment Trial (ONTT) [2]. The ONTT demonstrated the benefits of corticosteroids for speeding up short-term visual recovery although subsequent meta-analyses have demonstrated no significant visual improvement at the six-month and one-year follow-up when compared to placebo [2-3]. Alternative acute immunomodulatory therapies and plasma exchange therapy are considered after corticosteroid therapy failure [4]. There are no randomized controlled trials exploring the efficacy of plasma exchange in idiopathic optic neuritis. We present the case of a 43-year-old deaf and mute woman with recurrent idiopathic bilateral optic neuritis who experienced visual improvement with concurrent corticosteroids and early plasma exchange in order to preserve her vision and ability to communicate via American Sign Language (ASL).

## Case presentation

A 43-year-old female presented to the emergency department with a four-day history of headaches and blurry vision. Her past medical history was significant for well-controlled non-insulin-dependent diabetes, hypertension, and dyslipidemia. Notably, the patient was deaf and mute, and her primary means of communication was ASL. The headache quickly resolved with non-steroidal anti-inflammatory medication; however, the patient’s visual deficits remained.

Physical exam was notable for papilledema and several dot hemorrhages on dilated fundoscopic examination. The patient had a loss of visual fields in all quadrants bilaterally. Her visual acuity was estimated at 20/50 bilaterally. Pupils were equally round and reactive bilaterally to light and accommodation with no afferent pupillary defect. Eye alignment, eye movement, slit lamp examination, and intraocular pressures were normal. The patient’s vital signs were stable.

Non-contrast computed tomography (CT) and magnetic resonance imaging (MRI) with and without contrast of the brain demonstrated no abnormalities and no lesions or plaques. On suspicion of idiopathic intracranial hypertension due to her papilledema, a lumbar puncture was performed, and she was found to have an increased opening pressure of 32 mmHg. The patient was initially treated with an acetazolamide dose of 500 mg.

**Figure 1:**
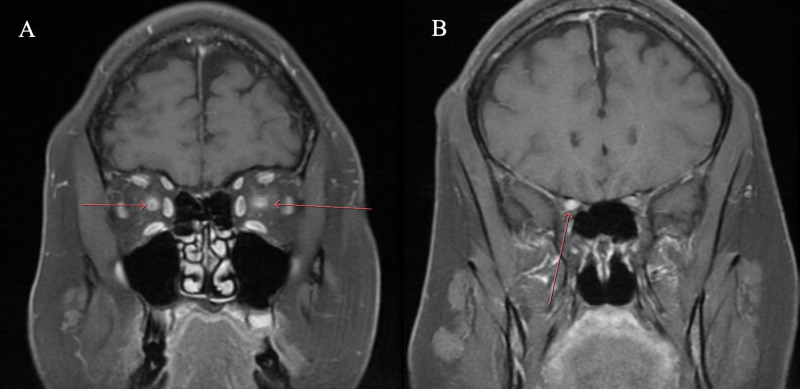
Patient’s initial presentation The optic nerves are edematous and enlarged. T1 post-gadolinium contrast, fat-suppressed, coronal view of the orbits demonstrating bilateral intraconal optic nerve enhancement (A) with slight extension into the right prechiasmatic optic nerve (B).

Contrast-enhanced MRI of the orbits revealed bilateral optic neuritis (Figure 1). Contrast-enhanced MRI of the cervical and thoracic spine showed no abnormalities and no lesions or areas of demyelination. The patient had a normal complete blood count and differential and a normal complete metabolic panel. Further lab testing (reference range in parentheses) revealed a normal thyroid-stimulating hormone (TSH) of 0.710 µIU/mL (0.38-5.33 µIU/mL), well-controlled diabetes with hemoglobin A1C of 6.6% (< 5.6%), slightly elevated high sensitivity C-reactive protein (CRP) at 11 mg/L (< 7.5 mg/L), and normal erythrocyte sedimentation rate (ESR) at 14 mm/hour (< 20 mm/hour). A toxicology panel checking for arsenic, lead, mercury, and cadmium levels was normal.

However, while waiting for blood cultures and other laboratory investigations, the patient’s vision worsened to the point that she was no longer able to communicate with health care staff via ASL. Given the unique situation necessitating vision for communication in this deaf and mute patient, we began a five-day course of intravenous (IV) methylprednisolone along with concurrent plasma exchange. After two treatments of plasma exchange and three days of corticosteroids, our patient’s vision markedly improved.

Autoimmune, demyelinating, metabolic, and infectious causes, including multiple sclerosis, neuromyelitis optica, lupus, connective tissue disorders, toxoplasmosis, West Nile virus, syphilis, and Herpes simplex virus (HSV), were ruled out via blood and cerebrospinal fluid (CSF) cultures. The CSF analysis was negative for oligoclonal bands, aquaporin 4 receptor, myelin oligodendrocyte glycoprotein antibodies, West Nile Virus antibodies, toxoplasmosis antibodies, and human immunodeficiency virus (HIV). Blood and urine cultures remained negative. HSV blood levels, rapid plasma reagin, and a comprehensive autoimmune panel were all normal. Of note, her Epstein-Barr virus (EBV) immunoglobulin G (IgG) antibodies to viral capsid antigen, early D antigen, and nuclear antigen were all markedly elevated. The patient was started on acyclovir given literature citing its possible effectiveness in optic neuritis secondary to EBV [5]. The patient completed five sessions of plasma exchange and continued to improve throughout the hospital course. She was discharged home in stable condition and set up to see outpatient neurology.

**Figure 2:**
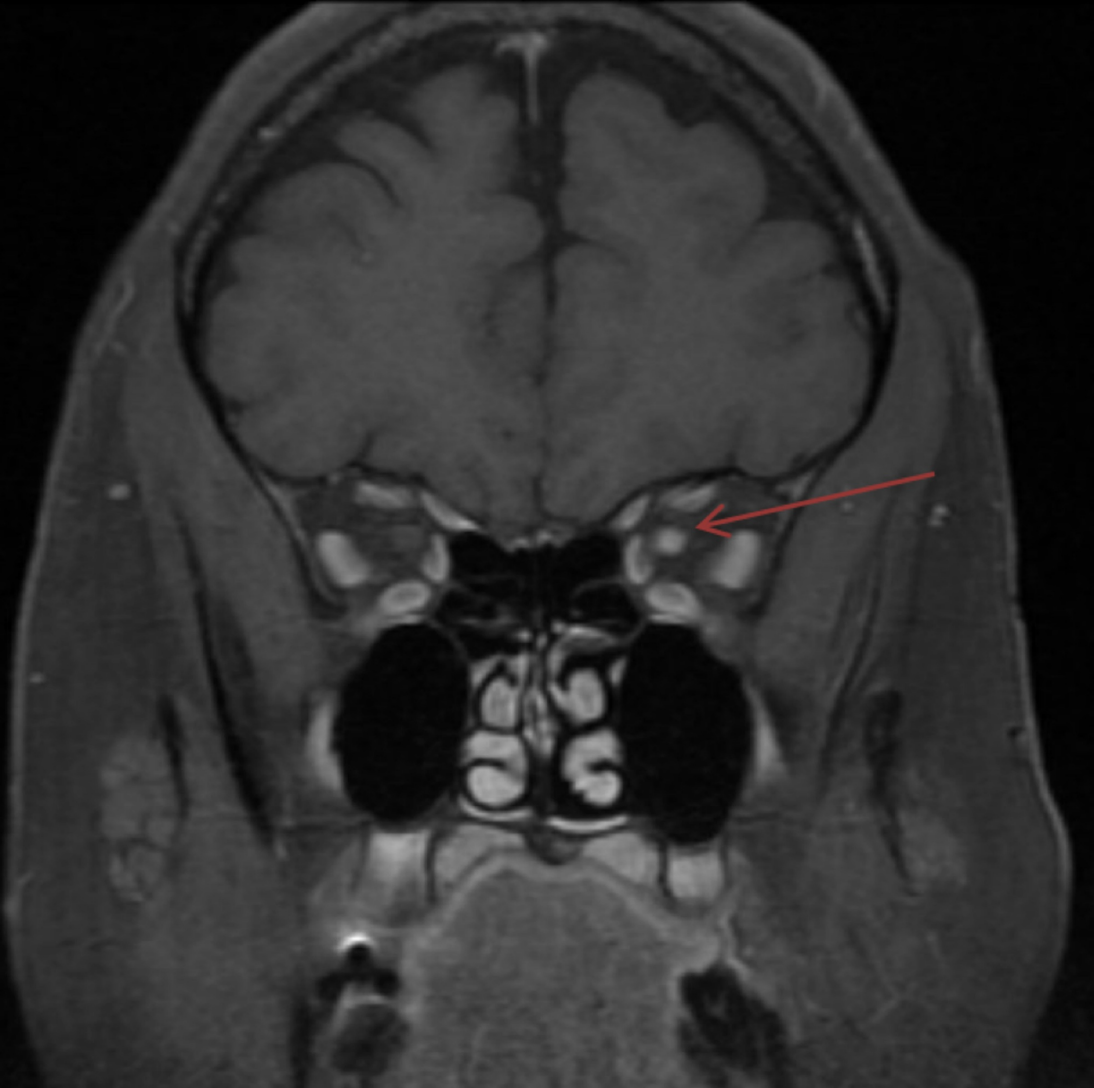
Patient’s presentation six months later T1 post-gadolinium contrast, fat-suppressed, coronal view of the orbits demonstrates increased enhancement of the left optic nerve head to the orbital apex consistent with optic neuritis. The previously seen enhancement in the right optic nerve has nearly completely resolved with a questionable tiny area of enhancement seen for which mild recurrent right optic neuritis is not excluded. There is persistent T2 hyperintensity within the optic nerves.

Unfortunately, the patient was lost to follow-up. She then presented to the emergency department six months later with similar symptoms, including headache and a decrease in visual acuity bilaterally. Her contrast-enhanced MRI again demonstrated optic neuritis, worse in the left than right (Figure 2). She was quickly started on her previous treatment regimen of concurrent five doses of plasma exchange and five days of IV methylprednisolone. Similar tests were again conducted at this second hospital visit which remained negative. She was discharged again with improved vision.

## Discussion

Our patient had a case of idiopathic bilateral recurrent optic neuritis. Although it is possible that her past EBV infection precipitated her optic neuritis, optic manifestations of EBV are typically mild and rarely reported in the literature. Additionally, optic neuritis secondary to EBV most often follows infectious mononucleosis (IM) [5]. Our patient had no history of IM recently; thus, it was an unlikely cause of her optic neuritis, especially after her recurrent episode. However, as the underlying etiology of her optic neuritis was unclear, she was administered acyclovir initially, which was later discontinued.

There is little data on idiopathic optic neuritis and optic neuritis that is seronegative and not associated with multiple sclerosis (MS) [6]. This idiopathic form of optic neuritis can be severe and in one of the larger studies done on a cohort of 23 patients with idiopathic optic neuritis, visual acuity prognosis was poor and the recurrence rate was 50% [6]. A shortcoming of treatment recommendations for optic neuritis is that regardless of the severity of symptoms or cause (unless infectious), the recommended treatment for cases of optic neuritis remains high-dose IV corticosteroids [2]. Alternative treatment modalities are reserved for corticosteroid failure. Alternative treatments include intravenous immunoglobin (IVIG) and interferon; however, studies have yet to show a significant benefit [1]. Interestingly, oral corticosteroids may be associated with an increase in optic neuritis recurrence [3,7]. While typical optic neuritis responds well to this treatment, there is little data regarding atypical presentation, such as our patient.

Only a handful of studies involving small cohorts have investigated treating severe optic neuritis refractory to corticosteroids with plasma exchange, and they have demonstrated significant improvement in visual acuity. Patients in these studies received plasma exchange on average 20 days after onset of symptoms and typically after two failed rounds of high-dose corticosteroids [8-9]. Ruprecht et al. reported that plasma exchange improved vision in seven out of 10 patients with optic neuritis secondary to MS who were all refractory to steroid treatment. The median number of days from symptom onset to initiation of plasma exchange was 34.5 days and patients received an average of five plasma exchange treatments [10]. However, this was a retrospective observational series, and the authors could not exclude the fact that the patients may have improved without plasma exchange, which was a limitation of this study. Interestingly, randomized double-blinded trials comparing IVIG to placebo in the treatment of acute optic neuritis demonstrated no effect on long-term visual acuity [11-12].

In this case presentation, the onset to initiating plasma exchange was two days following the visual decline, and the patient experienced rapid improvement in symptoms. Her improved vision enabled her to communicate, interact, and participate in her care. Waiting for the failure of corticosteroids may have resulted in worse visual acuity outcomes, protracted hospital stay, and increased emotional and physical stress for the patient. Clinical risks and benefits were carefully considered in this course of treatment. While not well-studied in the treatment of optic neuritis, plasma exchange is a generally safe and well-tolerated treatment [13]. Our patient’s need for visual acuity improvement weighed heavily in the decision to pursue early, aggressive treatment with plasma exchange. We recommend considering concurrent plasmapheresis and corticosteroids for the treatment of optic neuritis in patients whose symptoms are very severe or who have other functional limitations necessitating vision for communication. Our case is limited by its singular nature; however, future studies that investigate the early use of plasmapheresis and corticosteroids would be able to further elucidate this treatment regimen as a viable option for patients presenting similarly with idiopathic optic neuritis.

Our case demonstrates the importance of recognizing the cultural gaps, communication barriers, and health literacy issues associated with a deaf and mute patient. Deaf patients are less knowledgeable about health issues due to poor access to deaf-tailored information about disease processes [14]. This patient was lost to follow-up and had a recurrent event that affected a vital aspect of her life and daily function. Recognizing and mitigating these barriers are crucial to ensure adequate care and follow-up in the future and will be important in the future outcome of this patient.

## Conclusions

More research is needed in alternative therapies for optic neuritis, as the current accepted therapies are limited to corticosteroids, which have not been shown to have long-term benefits. Plasma exchange therapy used concurrently with corticosteroids yielded rapid improvement in symptoms for a case of idiopathic optic neuritis in a deaf and mute patient whose means of communication was ASL. Although further research is required, we suggest that concurrent treatment of corticosteroids and plasma exchange in the early stage of optic neuritis presentation may be beneficial, especially in patients with functional limitations that necessitate vision for communication
